# Menstruation hygiene management among secondary school students of Chitwan, Nepal:a cross-sectional study

**DOI:** 10.1186/s12905-023-02494-x

**Published:** 2023-07-26

**Authors:** Gayatri Khanal, Niki Shrestha, Kishor Adhikari, Usha Ghimire

**Affiliations:** Department of School of Public Health and Community Medicine, Chitwan Medical College Teaching Hospital (CMC), Bharatpur-13, P.O. Box: - 42, Chitwan, Nepal

**Keywords:** Menstruation, Hygiene Management, School girl, Secondary school, Chitwan

## Abstract

**Background:**

Despite significant progress in reproductive health, many societies still treat menstruation as disgraceful and shameful process and relate it with negative consequences. This belief and attitude may increase the incidence of reproductive tract infection, leading to significant negative impact on women’s health. To manage menstruation hygienically and with dignity, it is essential that women and adolescent girls have sufficient knowledge on menstruation hygiene management. Thus, this study aims to identify the knowledge, associated factors related to menstrual hygiene management (MHM) and effectiveness of health education on MHM.

**Methods:**

A school-based study was conducted between August 2021 to April 2022 among 400 secondary school girls. The sampling unit was selected by using systematic random sampling method. Health education was given in the interval between the pretest and posttest of knowledge assessment on MHM. Logistic regression analysis and Wilcoxon rank test were applied to identify predictors and to evaluate the effectiveness of health education.

**Results:**

Overall, 57.7% of the girls had unsatisfactory level of knowledge. Around two third (61.4%) missed school days during the menstrual period. Almost 99.5% had experienced some form of cultural restrictions during the menstrual period. After imparting health education, the level of knowledge on menstruation hygiene had significantly improved (z = 17.129, p = < 0.001) to satisfactory compared to the baseline knowledge level (42.3% vs. 92.5%). During multivariate analysis, factors such as having studied in public schools (AoR = 1.7, p = 0.026), having no or one close female friend (AoR = 2.2, p = 0.011), caste other than Brahmin/Chhetri (AoR = 1.4, p = 0.05) and factors such as living in joint family (AoR = 1.6, p = 0.048) were significantly associated with unsatisfactory level of knowledge on MHM.

**Conclusions:**

A substantial number of respondents had unsatisfactory level of knowledge on MHM. Factors such as ethnicity status, types of family, number of close female friends, types of schools and mothers’ education were associated with unsatisfactory level of knowledge. School absenteeism and cultural restriction were found to be common/frequent. Imparting of health education was found to be an effective measure to enhance knowledge on MHM.

**Supplementary Information:**

The online version contains supplementary material available at 10.1186/s12905-023-02494-x.

## Introduction

Menstruation is a natural process and a key sign of reproductive health. However, in many societies, it is treated as something negative, shameful or dirty. The continued silence around menstruation combined with limited access to information at home and in schools results in millions of women and girls having very little knowledge about what is happening to their bodies when they menstruate and how to deal with it [1, 2]. A study revealed that one out of three girls in South Asia knew nothing about menstruation prior to getting it [1].

Globally, approximately 52% of the female population (26% of the total population) is within reproductive age [3]. It is estimated that, 10% of women worldwide are exposed to genital infections including urinary tract infections and bacterial vaginosis, and 75% of women have a history of a genital infection due to pregnancy, poor perineal hygiene and poor menstrual hygiene [4]. To manage menstruation hygienically and with dignity, it is essential that women and adolescent girls have sufficient knowledge on menstruation hygiene management (MHM).3Recent evidences have reported that factors such as females being aware of menstrual hygiene and staying longer in schools has been associated with reduced maternal death; improved population health; increased contraceptive usage; decreased fertility rate, improved child health and decreased reproductive tract infections [5–9].

Every day, approximately 2, 90,000 women and girls in Nepal experience menstruation. With 82% of Nepalese women living in rural conditions, use of archaic, unhygienic, unhealthy and possibly hazardous menstrual hygiene management methods pushes Nepalese women deeper into the crevice of marginalization and reproductive health morbidity due to lack of adequate knowledge on MHM [8, 10]. Many girls and women face challenges managing their periods safely because of persisting taboos. For example, the custom of “Chhaupadi” is still prevalent in Nepal – a deplorable custom which relegates women to live in a cow shed and experience grossly unhygienic conditions while menstruating [2]. Adolescent girls often lack appropriate information about their reproductive health and proper menstrual management in low resource settings like Nepal. This in turn, has a direct impact on daily living, leading to school absenteeism as high as 53% in Nepal, affecting the girl child’s academic career [10], which could have been averted by raising awareness through health education [7–10].

Previous evidences from various studies conducted in different districts of Nepal have highlighted the presence of low awareness regarding menstruation hygiene [11–14]. Nepal government has achieved significant progress towards reproductive health in terms of maternal mortality and morbidity reduction. However, adolescent girls still face many sexual and reproductive health problems and challenges, amidst gender discrimination and disparity in Nepalese norms [8, 9].

Although literature on various aspects of menstruation hygiene is available to young girls, only negligible research has been done to investigate the associated factors and effectiveness of health education on enhancing the knowledge on MHM. Moreover, adolescent health and maternal health is one of the priority programs in Nepal. Thus, this study was conducted to identify the knowledge on MHM and its associated factors to assess the effectiveness of a health education program on enhancing the knowledge of MHM.

## Methods

### Study design, study settings and study participants

A school based cross-sectional study was conducted from May 2021 to April 2022 among secondary school girls in Chitwan District, Nepal. Seclusion and exclusion practices were found to be widespread, particularly among Brahmin, Chhetri and Newar castes who predominantly reside in Chitwan [15]. Secondly, Chitwan district was selected to represent different terrains of rural –urban areas [16]. In Chitwan district, there were 250 secondary schools, out of which 122 schools were public schools and 128 were private. There were 13,652 girls enrolled in the 9th and 10th grade for the academic year 2020–2021 out of which 9,344 were in public schools and 4,308 in private schools [17]. The girls who had attained menarche were included in the study, while those who were absent on the day of data collection were excluded from the study.

### Sample size and sampling procedure

The sample size was determined using population proportion formula given by Cochran with the assumption of 95% confidence, 5% allowable error, and prevalence of knowledge about menstruation hygiene being at 39.2% [18]. To compensate for the non-response rate, 10% of the determined sample was added up to the calculated sample size thus resulting with 400 final sample size.

The sampling procedure was started with the selection of the schools and then with the selection of the respondents from those selected schools. In the first round, 20 out of 250 secondary schools were selected by using a lottery method. For the lottery method, the names of all the secondary schools of the district were written in a separate, uniform-sized paper which was folded and put into a box. The papers were thoroughly mixed. One by one, the papers were taken out while the box was thoroughly shaken every time.

In the second round, the total list of respondents (9th and 10th grade girls) in each secondary schools were obtained from the student registration books of the respective schools. There were 1,237 girls in the selected schools, out of which 400 respondents were selected by using systematic random sampling procedure. To perform systematic sampling, kth items was calculated (1237/400 = 3.09th) and first number was assigned through simple random sampling techniques. Number of participants selected from each school is available in Additional file. If the selected respondent was absent on the day of the data collection, the next nearest was chosen as a respondent.

### Data collection tools and techniques

The data collection tool was semi-structured self-administered questionnaire. It was prepared in Nepali language based on the review of the relevant literatures. The tool was developed with the help of an extensive literature review of previous similar studies [11, 19, 20] and with assistance of expertise in the field of MHM. The data collection was done from 7th April to 27th April 2021.

Prior to the data collection, the respondents were approached through school authority. The respondents were clearly informed about the type of research and the objectives and goals of the research. Four of the final year students of Bachelor of Public Health were recruited as data collectors. They were given one day of training and education to familiarize with the study, and were told to maintain confidentiality of information. They were also told to collect data as per the research protocol. The supervision of data collection was done by the research team. In the first stage, the self-administered questionnaire was given as a pretest. In the second stage, health education was given and finally in the third stage, the posttest was carried out. The pretest questionnaire covered informations on demography, knowledge and practices related to MHM. The posttest questionnaire covered only knowledge related aspects. The posttest was done immediately after completion of the health education program which would likely decrease the chance of drop out. The health education was provided for a duration of 90 min which included the menstrual components like definition, causes, sources of bleeding, average duration of blood flow, normal menstrual cycle, pad use, handwashing practices and disposal of menstrual pad. The health education was delivered by using PowerPoint presentations, pamphlets and a short video. The education package was formulated with adequate literature review [21–25] and also with the aid of the expert in the area of MHM. To ensure the reliability of the tool and the educational package, pretesting was done. The pretesting was conducted among 10% of sample size in 4 high schools (2 public and 2 private schools) of Chitwan district to identify the reliability. Necessary modifications and improvisation to the questionnaire and health education package were done following the pretest.

### Data analysis

Data entry was done using Epi-data version 4.6. The data was checked for completeness and accuracy before feeding data on Epi-data 3.1. Data analysis was done using IBM Statistical Package for the Social Sciences (SPSS) Version 20. The statistical analysis carried out were the descriptive, bivariate (Wilcoxon sign rank test) and the multivariate analysis (Logistic regression). Those variables at p value ≤ 0.05 was considered as significant. Before multivariate analysis, the bivariate analysis was performed between dependent variable (level of knowledge) and each of the independent variables (socio-demographic and other variables), one at a time. The variables which were found significant at bivariate test were channeled into multivariate analysis using a logistic regression model in order to control for confounding factors.

The model for goodness of fit was checked using the Hosmer-Lemeshow test (p > 0.05.) and Variation Inflation Factors (VIF) of all significant independent variables which lies in the range of 1–4. The level of knowledge was calculated out of the 24 knowledge specific questions. Each correct response was considered as1 point and an incorrect response was considered as 0 point. The highest sum score of knowledge was 24 whereas lowest was 0. The level of knowledge was categorized asper the study conducted by Chetkanta et al., in Dang, Nepal [12]. Unsatisfactory level of knowledge was categorizes as a score of 0–12. Satisfactory level of knowledge was categorized as a score of 13–24.

#### Inclusion and exclusion criteria

Only those respondents who were studying in 9th or 10th grades at the time of data collection, were included in the study. Those respondents who hadn’t experienced menarche or who refused to provide informed consent and those who were absent on the day of the data collection were excluded from the study.

## Results

### Socio-demographic characteristics of the study participants

The respondents’ ages were between 12 and 19 years, with a mean age 15 and SD of ± 1. 87.3% of the respondents belonged to Hindu religion. Of the total, 50.7% were studying in public schools. Most of the schools (95.8%) were located near the market areas. 82.2% respondents had more than one close friend. 12.5% of the mothers of the respondents were illiterate.

### Menstruation related information

More than half (53.2%) of the respondents had never participated in MHM related program. The majority of the respondents (85%) had heard about menstruation before the menarche age. Nearly one-third (73.5%) of the respondents had experienced menarche at the age of 10–13 years. During the menarche, 204(51%) were scared, 73(18.2%) had cried, 68(17%) were embarrassed and only 13.8% were happy. 249 (63.1%) of the respondents experienced pre-menstrual and menstrual problems. 242 (61.4%) of the respondents had missed their schools due to menstruation. Out of them, 54.9%,14.4% and13.1% had missed one day, a couple of days and more than two days of school in each menstrual cycle. 74 (18.7%) of the respondents did not wash their hands after changing pads.

**Table 1:** Knowledge regarding MHM among secondary school girls of Chitwan. (n = 400)

Statements	Before health education (correct responses		After health education (correct responses)	
	Frequency	(%)	Frequency	(%)
Menstruation is the normal and healthy process in female	210	52.5	358	89.5
Menstruation cycle is related to pregnancy	376	94	390	97.5
Hormone is the main cause of female menstruation	323	80.8	398	99.5
Uterus is the main source of bleeding during the menstruation days	152	38	343	85.8
Normal duration of menstruation flow is 3–5 days	246	61.5	339	84.8
Normal menstruation cycle for healthy women is 28 days	210	52.5	353	88.3
10–15 years is average age of menarche	372	93	399	99.8
To manage blood flow and to maintain hygiene is the main reason to use sanitary pad during menstruation	393	98.3	393	98.3
In every 4–6 h, sanitary pads should be changed	311	77.8	387	96.8
Handwashing after pad change is a good practice	394	98.5	399	99.8
Burying and burning is the proper way to dispose a used pad.	76	19	205	51.3

**Table 2:** Knowledge regarding MHM among secondary school girls of Chitwan. (n = 400)

Statements	Before health education (correct responses		After health education (correct responses	
	Frequency	(%)	Frequency	(%)
Common problems faced before Menarche * Multiple response possible				
Headache	83	20.8	239	59.8
Constipation/diarrhoea	25	6.3	140	35
Abdominal bloating	106	26.5	208	52
Breast tenderness	181	45.3	217	54.4
Mood swings	140	35	198	49.5
Common problems faced during menstruation * Multiple response possible				
Cramp, back pain and discomfort	289	72.3	359	89.8
Loss of appetite, headache and tired	99	24.8	258	64.5
Full of abdomen and /or difficult to walk	167	41.8	233	58.3
Remedial measures for problems during menstruation, * Multiple response possible				
Maintain personal Hygiene	147	36.8	254	63.5
Take rest	260	65	308	77
Healthy diet	219	54.8	270	67.5
Light exercise	110	27.5	230	57.5
Take medicine	27	6.8	199	49.8

Tables 1 and 2 present the frequency and percentage of the knowledge based on the before (pre) and after(post) health education. Although there is a remarkable change seen in the level of knowledge in the after-health education as compared to that in the before health education, none of the respondents had scored a 100% correct response in both the before and after education sessions.

Before health education, 38% of the total respondents were aware of uterus as the only source of menstrual bleeding; 52.5% knew menstruation was normal and healthy process and 19% admitted burying or burning was the correct way to safely dispose the sanitary pads. Breast tenderness (45.3%), mood swings (35%), bloating (26.5%), headache (20.8%) and constipation or diarrhea (6.3%) were the common problems faced by the girls before the onset of menstruation. Loss of appetite, headache and tiredness (24.8%), fullness of abdomen and/or, difficulty in walking (41.8%) were the common problem faced during menstruation. Maintaining personal hygiene (36.8%), performing light exercises (27.5%) and taking medicine if necessary (6.8%) were the remedial techniques followed to deal with the problems during menstruation.

Despite an overall improvement of knowledge in after health education, knowledge on correct way (burying or burning) of disposing pads (51.3%) and knowledge on commonest problems faced during menstruation (constipation/diarrheas (36%), bloating (52%), mood swings (49.5%) and breast tenderness (54.4%) was not expectantly increased.

**Table 3:** Level of knowledge regarding MHM among secondary school students, Chitwan. (n = 400)

Time	Level of knowledge	n/%
Before health education	Unsatisfactory	231/57.7
	Satisfactory	169/42.3
After health education	Unsatisfactory	30/7.5
	Satisfactory	370/92.5

Table 3 compares about the level of knowledge in before and after health education. The unsatisfactory level of knowledge among the respondents had decreased from 57.7% (pretest) to 7.5% (posttest) while there was a significant improvement in satisfactory level of knowledge (from 42.3 to 92.5%) in before and after health education. This data clearly explained that the satisfactory level of knowledge had improved after menstrual education intervention on MHM.

**Table 4:** Logistic regression analysis for factors associated with MHM knowledge among secondary school girls of Chitwan (n = 400)

Variables	COR (95% CI)	P-value	AOR (95% CI)	P-value
Ethnicity				
Brahmin/Chhetri	1			
Other than Brahmin/Chhetri	1.71	0.014#	1.447	0.05#
Type of family				
Nuclear	1			
Joint	1.54	0.055#	1.604	0.048#
Type of school				
Public	2.18	0.0001#	1.742	0.026#
Private	1			
Father occupation				
Agriculture	0.794	0.405	0.718	0.289
Business/service	0.52	0.008#	0.702	0.201
Others (daily wages,)	1			
Mother education				
Illiterate	2.14	0.029#	0.903	0.806
Primary/secondary	1.76	0.013#	1.080	0.770
Higher secondary and above	1			
Mother occupation				
Agriculture	0.933	0.814	0.920	0.797
Business/service	0.55	0.045#	0.609	0.123
Others	1			
Close female friend				
1 or no friend	2.5	0.002#	2.196	0.011#
More than 1	1			
Number of sisters				
No or one sister	1			
More than one sister	1.65	0.033#	1.276	0.335

(Adjusted with ethnicity status, Types of family, Types of schools, Father Occupation, Mother Occupation, Mother Education, Number of close friend and number of sisters) (1) # denotes statistically significant association, (2) Hosmer and lemeshow test p = 0.583, (3) Cox and snellR.Square = 0.092, (4) Nagelkerke R. Square = 0.123, 4. 1 = Reference category

The bivariate logistic regression was used to examine any factors that may be important with knowledge on menstruation hygiene management. The bivariate analysis showed that girls with ethnicity otherthan Brahmin/Chhetri(OR = 1.7, p = 0.014), living in joint family (OR = 1.5, p = 0.055), studying in public school (OR = 2.18, p = 0.0001), primary or secondary level mother’s education (OR = 1.7 p = 0.013), illiteracy (OR = 2.14 p = 0.029), having no or one close female friend (OR = 2.5 p = 0.002) and having more than one sister (OR = 1.65 p = 0.033) were more likely to be the factors associated with the unsatisfactory level of knowledge on MHM. In case the respondents’ father OR = 0.52 p = 0.008) and mother occupation (OR = 0.55 p = 0.045) was business or service, they were possibly less likely to have unsatisfactory level of knowledge on MHM (Table 4).

Multiple logistic regression was used for multivariable analysis to get the final model in this study. Those variables which were found statistically significant (p < = 0.05) in bivariate analysis, were included into the multivariable regression analysis. The findings revealed that respondents whose ethnicity was other than Brahmin/Chhetri (AoR = 1.4, p = 0.05), lived in joint family (AoR = 1.6, p = 0.048), studied in public school (AoR = 1.7, p = 0.026), had one or no close female friend (AoR = 2.2, p = 0.011) were significantly associated with unsatisfactory level of knowledge as presented in Table 4.

**Table 5:** Before and after knowledge regarding MHM among secondary school student of Chitwan. (n = 400)

Knowledge on MHM	(Q1, Q2, Q3)	Min/Max	Sum rank (-/+)	P-value
Before	(10,12,14)	6/23	41 / 76,204	< 0.0001##
After	(15,18,20)	8/24		

## denotes two tail significance (by applying Wilcoxon sign rank test at 5% level of significance with z = 17.129)

Table 5 showed that the level of knowledge rank in after health education was statistically significant and higher than level of knowledge rank in before health education (z = 17.129, p = < 0.001). This result clearly explained that the providing education on menstruation hygiene to the secondary school girls is one of the effective strategies to enhance level of knowledge on menstruation hygiene. Most of the participants (90.5%) had experienced cultural restrictions during menstruation. The commonest limitations found during menstruation were for visiting temple (97%), performing household rituals/puja (95.3%), attending religious functions (88%), and for cooking in kitchen (64.2%). The least limited activity was for eating food and drinks (18.5%). (Table 6)

**Table 6:** Cultural restriction faced during menstruation. (n = 400)

Variables	Frequency	Percentage (%)
Ever experienced cultural restriction during menstruation		
Yes	362	90.5
No	38	9.5
If yes which #		
Visit temple	359	97
Attend religious function	352	88
Perform household puja	346	95.3
Touch male family member	127	35
Cook food or enter inside kitchen	233	64.2
Go outside as much as normal	76	20.9
Eat food or drinks of their choice	67	18.5
Sleep in the same bed with others	85	23.4
Sleep in the household members as others	144	39.7

# denotes multiple response

## Discussion

This study aimed to assess the level of knowledge on MHM, it’s associated factors and the effectiveness of health education regarding MHM among the respondents. In the above analysis, we found a towering percentage of unsatisfactory level of knowledge on MHM. Having lesser number of close friends, engaged to public schools, belonging to castes other than Brahmin/Chhetri caste and living in the joint family was positively associated with an unsatisfactory level of knowledge. A large number of respondents were found to have missed school days during menstruation and were also found to face multiple cultural restrictions during menstruation. Significant changes on the level of knowledge were observed after delivering the health education on MHM.

The findings of this study revealed that the more than half (57.7%) of the respondents had unsatisfactory level of knowledge regarding MHM which is comparable with various studies conducted in Kathmandu, Nepal (60%), Northeast, Ethiopia (49%) and Oyo state, Nigeria (44.1%) [11, 19, 20]. The reasons behind the consistency could possibly due to fact that all countries are labelled as low- and middle-income countries and therefore, the constraints faced by the girls regarding MHM may be similar. The study done in Dang, Nepal (22.3%), in Amhare, Ethiopia (9.3%), in Sokoto, Nigeria (35%) and in South-western Nigeria (30%) found that a lower proportion of the respondents had unsatisfactory or lower level of knowledge, respectively [12, 26–28]. In contrast to this study, the study done in Doti, Nepal (73.6%), in Araihazar, Bangladesh (72.2%), in Baghdad, Iraq (74%), in Riyan City, Saudi (75.9%) had revealed a higher proportion of girls with unsatisfactory knowledge on MHM [13, 29–31]. The differences might be due to changes in time and improvised & updated provision of educational materials as compared to previous times. The differences might also be because of divergence scoring system and the inclusion of other several numbers of questions related to knowledge assessment for measuring the knowledge level of MHM in different studies.

This study revealed that, girls who were studying in private schools were more likely to have satisfactory level of knowledge in comparison to the those studying in public schools which is in line with the study conducted in Dang, Nepal [12]. A study conducted in northwestern Nigeria reported a contradictory result with the type of school which was found to be insignificant regarding knowledge of menstruation [32]. The difference might be due to the differences in school policy/norms of different country settings.

This study also revealed that having a greater number of close female friends determine the satisfactory level of knowledge on MHM. This finding is similar to the findings of the study in Jodhpur India where peer educators or friends were regarded as an effective method of enhancing awareness among adolescents on menstruation hygiene [33]. Similarly, the study conducted in Damietta City, Egypt revealed that adolescents girls are highly influenced by peer and they are very likely to listen and follow what their peers say [23].

Respondents who were living in a joint family are most likely to have unsatisfactory level of knowledge as compared to those living in nuclear family. This could be due to the fact that increased chances of better interactions and emotional bonding with mothers and sisters along with enhanced privacy is ensured with the nuclear family as compared to the joint state. Similarly, educational status of the mothers was one of the predictors for level of knowledge among respondents. This finding is similar to the various reports of other studies conducted in Dang, Nepal [12], Amhare Ethiopia [26], Oyo, state, Nigeria [20], West Bangal, India [34], and in Kathmandu, Nepal [7]. Girls always feel safer and comfortable while discussing menstruation with their mothers. Illiteracy of the mother always imparts insufficient knowledge along with myths and taboos to the female child. Hence, mother’s education showed a significant relationship with the level of knowledge on MHM [35]. This study also highlighted that ethnicity status had a role in determining the knowledge on MHM. This observation was in agreement with other studies from West Bengal, India [34, 36] and from Uttar Pradesh, India [37]. This could be due to the fact that people from Nepal and India both share a similar context of Hinduism which is obviously reflected in caste and ethnicity.

This study revealed that, majority of the respondents (61.4%) missed school days due to menstruation. Similar findings were reported by Wash United on MHM day, which mentioned that 53% of the menstruating Nepali girls are absent in the school during the menstruation [10]. Prior to the health education, the respondents had extremely low level of knowledge on the following aspects: “Menstruation as normal and healthy process in female”, “Uterus as the main source of bleeding during the menstrual cycle”, “Normal menstruation cycle of 28 days”. This was supported by studies conducted in Araihazar, Bangladesh 38 and Oyo state, Nigeria [20]. However, a higher proportion of school girls reported correct responses to the aforementioned study variables as stated by several studies conducted in Dang Nepal [12], in Doti, Nepal [13] and in Chitwan, Nepal [14]. This difference might be due to the different study settings (urban vs rural), age selection of the participants (private schools vs public schools) and also the school environment and culture practices regarding menstruation hygiene management.

This study reveals that a predominant population of respondents experienced restriction to cultural activities during menstruation. The most commonly limited activity was attending and performing religious chants and activities. Similar findings were observed in various other studies conducted in West Bengal, India [34], Chitwan Nepal [14] and in Kathmandu Valley, Nepal [7]. Another study done in the rural areas of West Bengal elicited that restricting sour foods and disallowing to visit temple have been the most common practices during menstruation [36]. This finding is more or less consistent with the present study. Evidence throw light on existing social discriminations, deep–rooted cultural and religious superstitions among girls and women are prominent in Hindu religion which might lead to deteriorating reproductive health [7].

The present study demonstrates that level of knowledge regarding MHM was relatively low before (42.3%) but significantly high (82.4%) after the educational intervention. These findings are similar to the results of other studies done in Bangladesh (51% Vs 82.2%) [38], Korea (42% vs. 75%) [21] and Riyan City Saudi (38% vs. 65%), [31]. The varied increment proportion of knowledge level in the above findings may be due to factors like; duration and frequency health education, place chosen for health education, educational level of students, and previous exposure to health education program by the students. In this research, there was a significant difference (z = 17.13, p = < 0.001). in the level of knowledge ranks in the pre and post health educated participants. It corroborates the fact that education on menstruation hygiene to the secondary school adolescent girls is one of the effective strategies to enhance the level of knowledge on menstrual hygiene. These results are similar to the results of a study conducted in Southern Haryana, India [22], Riyan City Saudi [31], Damietta City, Egypt, [23] Jumla District, Nepal, [24] and in Pune city, India [25].

### Limitations

This study has revealed several crucial findings and insights regarding MHM for school going adolescent girls. Nevertheless, it also has some limitations. First, the posttest findings in this study were taken immediately after the health education intervention on MHM. Hence, the girls may have reported with better knowledge on MHM. Secondly, information about school absenteeism and cultural restrictions faced were based on self-reporting which may vary accordingly. Although all possible efforts were applied to standardize the educational intervention, it is possible that the individual and environmental factors such as differences in the abilities of data collectors and their ability to disseminate study messages may play a vital role. In spite of such constraints, the present study has made an effort to explore major findings on MHM, which may play a crucial role for policy makers in improvising reproductive health status of the girls and women in Chitwan, Nepal.

## Conclusion

The majority of the respondents had unsatisfactory level of knowledge on MHM. Knowledge on MHM was found to be associated with ethnicity, types of family, number of close female friends, types of schools and mothers’ education. All these factors should be taken into consideration while formulating plans, policies and programs regarding the MHM. School absenteeism and cultural restriction faced during menstruation was found to be substantially high. The health educational intervention was found to be an effective measure to enhance the knowledge on MHM. Regular consistent educational programs may be needed for sustainable knowledge and better practice in MHM.

## Electronic supplementary material

Below is the link to the electronic supplementary material.

Additional File 1: Questionnaire for participants

Additional File 2: Nepal Health Research Council

Additional File 3: Total number of participants selected from each selected school

## Data Availability

The dataset used during the study is included in this manuscript. [and its supplementary information files].

## References

[1] Mouse S, Mohan T, Cavill S. Menstruation hygiene matters:A resource for improving menstruation hygiene around the world.Dhaka, Bangladesh.Wateraid;2012. Available from: https://washmatters.wateraid.org/sites/g/files/jkxoof256/files/Menstrual%20hygiene%20matters%20low%20resolution.pdf.

[2] Amatya P, Ghimire S, Callahan KE, Baral BK, Poudel KC (2018). Practice and lived experience of menstrual exiles (Chhaupadi) among adolescent girls in far-western Nepal. PLoS ONE.

[3] Fast facts: nine things you did not know about menstruation [Internet]. Unicef; 2018 May25. Available from: https://www.unicef.org/press-releases/fast-facts-nine-things-you-didnt-know-about-menstruation.

[4] Reid G, Bruce AW (2003). Urogenital infections in women: can probiotics help?. Postgrad Med J.

[5] Dasgupta A, Sarar M (2008). Menstrual hygiene: how hygienic is the adolescent girl?. Indian J Community Med.

[6] Ten VA, Menstrual Hygiene: A Neglected Condition for the Achievement of Several Millennium Development Goals. European Commission- Europe Aid; 2007.1–24. Available from: https://www.ircwash.org/sites/default/files/Tjon-A-Ten-2007-Menstrual.pdf.

[7] Mukharjee A, Lama M, Khakurel U, Jha NA, Ajose F, Acharya S (2020). Perception and pratices of menstruation restrictions among urban adolescent girls and women in Nepal: a cross-sectional survey. BMC Reproductive Health.

[8] Water Aid. : Menstrual hygiene and management an issue for adolescent school girls. March 2009. Available from: http://www.wateraid.org/nepal.

[9] Morrison J, Basnet M, Bhatta A, Khimbanjar S, Joshi D, Baral S. Menstrual hygiene management in Udayapur and Sindhuli district of Nepal. Nepal. Wateraid; July 2016. 1–52. Available from: https://www.herd.org.np/uploads/frontend/Publications/PublicationsAttachments1/1484557187-Menstrual%20hygiene%20management%20in%20Sindhuli%20and%20Udaypur%20Nepal%5B1%5D.pdf.

[10] Public Health Update. Education about menstruation changes everything: Menstruation Hygiene Day [Internet]. Sagunblog; 2020 August 6. Available from: https://publichealthupdate.com/education-about-menstruation-changes-everything-menstrual-hygiene-day-mhday2017-menstruationmatters/.

[11] Adhikari B, Kandel SL (2007). .Dhungel, and AMandal. Knowledge and practice regarding menstrual hygiene in rural adolescent girls of Nepal. Kathmandu Univ Med J.

[12] Bhusal KC, Bhattarai S, Kafle R, Shrestha R, Chhetri P, Adhikari K (2020). Level and Associated factors of knowledge regarding Menstrual Hygiene among School- going adolescent girls in Dang District Nepal. Adv Prev Med.

[13] Yadav NR, Joshi S, Poudel R, Pandeya P (2018). Knowledge, attitude, and practice on menstrual hygiene management among school adolescents. J Nepal Health Res Counc.

[14] Neupane MS, Sharma K, Bista AP, Subedi S, Lamichhane S (2020). Knowledge on menstruation and menstrual hygiene practices among adolescent girls of selected schools of Chitwan. J Chitwn Med Coll.

[15] CBS. National Population and Housing Census. 2011. Social Characterstics Table (Caste/Ethnicity, Mother Tongue and Second Language). Kathamndu: National Planning Commission Secretariates, Central Bureau of Statistics; 2014.

[16] CBS (2012). Nepal in figures.

[17] Education Development and Coordination Unit, Chitwan, 2019.

[18] Upashe SP, Tekelab T, Mekonnen J (2015). Assessment of knowledge and practice of menstrual hygiene among high school girls on western Ethiopia. BMC Women’s health.

[19] Tegegne TK, Sisay MM (2014). Menstruation Hygiene Management and school absenteeism among female adolescent students in Northeast Ethiopia. BMC Public Health.

[20] Fehintola FO, Fehintola AO, Aremu AI, Ogunlaja A, Ogunlaja IP (2017). Assessment of knowledge, attitude and practice about menstruation and menstruation hygiene among secondary high school girls in Ogbomoso, Oyo state,Nigeria. Int J Reprod Contracept Obstet Gynecol.

[21] Min J, Ahn S (2018). Effects of menstrual self-management education program on knowledge and behavior of menstrual self-management in high school girls. Korean J women Health Nurs.

[22] Singh A, Gupta V, Agrawal D, Goyal P, Singh M, Lukhmana S (2020). A cross-sectional study to investigate the impact of focused group discussion on menstrual hygiene among rural school girls of Southern Haryana, India. J Educ Health Promotion Oct.

[23] Mohamad HA, El-Karmalawy EM, Gida NI, Hafez FE (2018). Effects of health education program on menstrual practices among secondary school girls. Port Said Scientific Journal of Nursing June.

[24] Dahal A, Acharya KP (2019). Effectiveness of information education and communication on menstrual hygiene among adolescent school girls of Jumla District. J Nobel Med Coll.

[25] Prema S, Dhandapani D, Prakash D, Gawade S (2020). Effectiveness of planned health teaching on knowledge and self –reported practices of menstrual hygiene among visually impaired adolescent girls in selected blind schools of Pune city. Int J Adv Nurs Manage.

[26] Gultie DH, Workineh Y (2014). Age of menarche and knowledge about menstrual hygiene management among adolescent school girls in Amhara province,Ethiopia:implecation to health care workers and school teachers. PLoS ONE.

[27] Oche MO, Umar AS, Gana GJ, Ango JT (2012). Menstrual health: the unmet needs of adolescent girls in Sokota, Nigeria. Sci Res Essays.

[28] Aluko OO, Oluya OM, Olaleye OA, Olajuyin AA, Olabintan TF, Oloruntoba-Oju OI (2014). Knowledge and menstrual hygiene practices among adolescent in senior secondary schools in Ile Ife, south-western. Nigeria J Water Sanitation Hygiene Dev.

[29] Haque ES, Rahman M, Itsuko,Mutaharu M, Sakisaka K (2014). The effect of a school-based educational intervention on menstrual health: an intervention study among adolescent girls in Bangladesh. BMJ Open.

[30] Sadiq AM, Salih AA (2013). Knowledge and practice of adolescent females about menstruation in Baghdad. J Gen Pract.

[31] AI Ayafi AS. Factors associated with health behavior regarding menstruation among Saudi intermediate school girls in Riyadh City (Doctoral Dissertation, Master thesis). Nursing College, King Saud University. 1999. Available on: https://scholar.google.com/scholar?q=Ayafi+SA++Factors+associated+with+health+behavior+regarding+menstruation+among+Saudi+intermediate+school+girls+in+Riyadh+city+++Master+thesis+.++Nursing+College,+King+Saud+University+,++1999+.

[32] Lawan UM, Yusuf NB, Musa AB (2010). Menstruation and menstruation hygiene amongst adolescent school girls in Kano, Northwestern Nigeria. Afr J Reprod Health.

[33] Dwivedi R, Sharm C, Bhardwaj P, Singh K, Joshi N, Shrama PP (2020). Effect of peer educator-PRAGATI (peer action for Group Awareness through intervention) on knowledge, attitude, and practice of menstrual hygiene in adolescent school girls. J Family Med Prim Care July.

[34] Sarkar I, Dobe M, Dasgupta A, Basu R, Shahbabu B (2017). Determinants of menstrual hygiene among school going adolescent girls in a rural area of West Bengal. J Family Med Prim Care.

[35] Omidyar S, Begum K (2010). Factors influencing hygienic practices during menses among girls from south India-a cross sectional study. Int J Collaborative Res Intern Med Public Health.

[36] Ray S, Dasgupta A (2012). Determinants of menstrual hygiene among adolescent girls:a multivariate analysis. Natl J Community Med.

[37] Malhotra A, Goli S, Coates S, Mosquera-Vasquez M (2016). Factors associated with knowledge, attitudes, and hygiene practices during menstruation among adolescent girls in Utter Pradesh. Waterlines July.

[38] Haque SE, Rahman M, Itsuko K, Mutahara M, Sakisaka K (2014). The effect of a school-based educational intervention on menstrual health: an intervention study among adolescent girls in Bangladesh. BMJ Open.

