# Supplementary material for: Menstruation hygiene management among secondary school students of Chitwan, Nepal:a cross-sectional study
Source: BMC Womens Health. 2023 Jul 26;23:395. doi: 10.1186/s12905-023-02494-x (PMC10373230; doi:10.1186/s12905-023-02494-x)
Supplement: Supplementary file 1 — Additional File 1: Questionnaire for participants [file 12905_2023_2494_MOESM1_ESM.docx]

**ANNEXES**

**Annex 1: Informed Consent form**

**Informed Consent form in English**

Questionnaire for participants

"**Menstruation hygiene management among high school adolescents of Chitwan, Nepal**’’

Introduction and permission

Namaste! My name is Gayatri Khanal. I am working in School of Public Health, Chitwan Mdical College, Tribhuwan University, Kailashnagar, Chitwan. I am conducting research on " **Menstruation hygiene management among high school adolescents of Chitwan, Nepal**. Since you will be the most knowledgeable in this area I want to ask you some questions which will take around 30 minutes and we will provide you relevant information regarding Menstruation hygiene. The information provided by you is expected to be useful for the Menstruation health program planning and implementation. This is to assure you that the information provided by you will be utilized for the study purpose only and will be confidential.

It is your decision to participate in the study. It is your right to withdraw from the study at any time. But I humbly request and hope for your support in this study.

If you have any questions regarding this study, you may ask ……………………………………

Are you interested to participate in the study?

Yes …………………………1

No ………………………….2

If yes, start interview

If no, greet and proceed for another respondent

**Personal information**

Id No of the participants. ……………………

Name of school ………………………..

Rural/Municipal name: ………………………

# Annex 1: Informed Assent form

**Informed assent form in English**

Questionnaire for participants

"**Menstruation hygiene management among high school adolescents of Chitwan, Nepal**’’

Introduction and permission

Namaste! My name is Gayatri Khanal. I am working in School of Public Health, Chitwan Mdical College, Tribhuwan University, Kailashnagar, Chitwan. I am conducting research on " **Menstruation hygiene management among high school adolescents of Chitwan, Nepal**. Since you will be the most knowledgeable in this area I want to ask you some questions which will take around 30 minutes and we will provide you relevant information regarding Menstruation hygiene. The information provided by you is expected to be useful for the Menstruation health program planning and implementation. This is to assure you that the information provided by you will be utilized for the study purpose only and will be confidential.

It is your decision to participate in the study. It is your right to withdraw from the study at any time. But I humbly request and hope for your support in this study.

If you have any questions regarding this study, you may ask ……………………………………

Are you interested to participate in the study?

Yes …………………………1

No ………………………….2

If yes, start interview

If no, greet and proceed for another respondent

**Personal information**

Id No of the participants. ……………………

Name of school ………………………..

Rural/Municipal name: ………………………

**SECTION I: Socio-Demographic information**

| **SN** | **Questions** | **Response Categories** | **Codes** | **Skip** |
| --- | --- | --- | --- | --- |
| 1 | Age of the respondent (completed years) | …… |  |  |
| 2 | Caste/ethnicity of the family  (As per HMIS) | Dalit  Janajati  Madeshi  Muslim  Brahmin/ Chhetri  Thakuri, sanyasi  Others(spe…..) | 1  2  3  4  5  6 |  |
| 3 | Religion of the family | Hindu………………  Buddhism………….  Muslim…………….  Christianity……….  Other (Specify)……. | 1  2  3  4  5 |  |
| 4 | Education(class/grade) of the student | …………. |  |  |
| 5 | Residence | Urban  Rural | 1  2 |  |
| 6 | Father's education | Illiterate……………  Literate  Primary…..  Secondary……  Higher Secondary  Bachelor and above…. | 1  2  3  4  5 |  |
| 7 | Mother’s education | Illiterate……………  Literate  Primary…..  Secondary……  Higher Secondary  Bachelor and above…. | 1  2  3  4  5 |  |
| 8 | Number of female siblings | One  More than one | 1  2 |  |
| 9 | Type of school | Public  Private | 1  2 |  |
| 10 | Locality of School | Near market or city  Far from market or city | 1  2 |  |
| 11 | Type of family | Nuclear………...  Joint/extended……. | 1  2 |  |
| 12 | Father’s Major occupation | Agriculture  Business  Service  Foreign employment...  Wage labour……..  Other specify… | 1  2  3  4  5  6 |  |
| 13 | Mother's Major occupation | Agriculture  Business  Service  Foreign employment...  Wage labour……..  Other specify… | 1  2  3  4  5  6 |  |
| 14 | Age at menarche (Age at the first menstruation) | 10-13  14-16  17-20 | 1  2  3 |  |
| 15 | Number of close female friends | None  One  More than 1 | 1  2  3 |  |
| 16 | Do you ever participate any MHM programme | Yes  No | 1  2 |  |
| 17 | Is your family monthly income is sufficient for food and daily activities? | Insufficient  Sufficient | 1  2 |  |

**SECTION II: Knowledge on MHM**

| **SN** | **Questions** | **Response Categories** | **Codes** | **Skip** |
| --- | --- | --- | --- | --- |
| 1 | Aware of menstruation before menarche | Yes  No | 1  2 | If yes |
| 2 | Source of information | Mother  Older sister  Older sister-in-law  Female relatives /Friends  Teachers  Mass Media (Books/magazines/health books ,Radio  Television) | 1  2  3  4  5  6 |  |
| 3 | Is the given information useful | Useful  Non-useful | 1  2 |  |
| 4 | Where were you when your first menstruation occurred? | At home  At school  Others(specify) | 1  2  3 |  |
| 5 | What was your first reaction when you experienced your first menstruation? | Cried  Scared  Embarrassed  Happy | 1  2  3  4 |  |
| 6 | What is menstruation? | Normal healthy process  Bad blood being shed  Others (curse, supernatural reason)  Don't know | 1  2  3  4  5 |  |
| 7 | Does Menstrual cycle is related to pregnancy? | Yes  No | 1  2 |  |
| 8 | Cause of menstruation | Hormonal  Diseases  Don't know | 1  2  3 |  |
| 9 | Source of bleeding | Uterus  Bladder  Vagina  Abdomin | 1  2  3  4 |  |
| 10 | Normal duration of menstrual flow | <3 days  3-5 days  >5 days | 1  2  3 |  |
| 11 | Normal menstruation cycle | 28 days  20 days  30 days  35 days |  |  |
| 12 | Reason to use sanitary pad | Manage blood flow & maintain Hygiene  To relieve pain  Instead of taking shower  Don’t know | 1  2  3  4 |  |
| 13 | Time interval to change pad | Every hour  Every 4-6 hours  Daily | 1  2  3 |  |
| 14 | Way to dispose of used pad | Burning  Burying  Throw away  Both a and b | 1  2  3  4 |  |
| 15 | Major Problems faced before onset of menstruation* Multiple response possible | Headache  Constipation/diarrhoes  Abdominal bloating  Breast tenderness  Mood swings | 1  2  3  4  5 |  |
| 16 | Problems faced during menstruation | Cramp, back pain and discomfort  Loss of appetite , headach and tired  Full of abdomen, difficult to walk | 1  2  3 |  |
| 17 | Remedial measures for problems  during menstruation* Multiple response possible | Maintain personal  Hygiene  Take rest  Drink lots of water / eat  Fruits  Exercise  Take medicines | 1  2  3  4  5 |  |

**SECTION III: Menstrual Hygiene Management Practice**

| **SN** | **Questions** | **Response Categories** | **Codes** | **Skip** |
| --- | --- | --- | --- | --- |
| 1 | Which material do you usually use to  Manage menstruation? | Cloths  Sanitary pad (disposable)  Don’t use any materials  Sanitary pad (reusable) | 1  2  3  4 |  |
| 2 | If you could choose one thing, what would you like best to manage your menstruation? | Sanitary pads (disposable)  Cloth (reusable)  Others( reusable sanitary pad, Tissue  Paper) | 1  2  3 |  |
| 3 | Shop within 20 minutes walking  distance of your home to buy  sanitary pad | Yes  No | 1  2 |  |
| 4 | Have ever used sanitary pads | Yes  No | 1  2 | If no move to question no 8 |
| 5 | If Yes, How often? | Usually  Sometimes  Rarely | 1  2  3 |  |
| 6 | Who bought it? | Self-bought  Relative bought | 1  2 |  |
| 7 | Where from | Bazaar(town) shop  local shop  others(got from school, medical shop ,  got with relief material) | 1  2  3 |  |
| 8 | Reasons for not using or rarely using  sanitary pads* (multiple response) | Not easily available  Cloths is more comfortable  Expensive  Unsure of how to use them  Embarrassed to go and buy them  Difficult to dispose of  Others (never heard of them, allergies of  pad) | 1  2  3  4  5  6  7  8 |  |
| 9 | When you are menstruating, how often  do you bathe?* multiple response | First day  Second day  Third day  Fourth day  Fifth day  Sixth day  Seventh day  Everyday | 1  2  3  4  5  6  7  8 |  |
| 10 | When you are menstruating, how often do you use soap to bathe? | Always  Sometimes  Never | 1  2  3 |  |
| 11 | When you are menstruating, how often do you able to wash genitals? | Always  Sometimes  Never | 1  2  3 |  |
| 12 | When you are menstruating, how much rest do you take? | More than usual  As normal  Less than normal  Don’t know | 1  2  3  4 |  |
| 13 | Do you maintain Privacy to wash reusable pads or cloth | Yes  No  Don’t use reusable cloths | 1  2  3 |  |
| 14 | Place to dry used cloth | Outside home in sunlight in the normal  place where clothes are dried  Outside home in the sunlight in a place  away from view  Outside home covered by other clothes  Others | 1  2  3  4 |  |
| 15 | Wash hand after changing pad | Yes  No | 1  2 |  |
| 16 | Areas of used pad disposal | Dustbin  Throw it in the field  Throw in Municipality garbage  Others (Specify)……… | 1  2  3  4 |  |
| 17 | Major Problems faced before onset of menstruation* Multiple response possible | Headache  Constipation/diarrhoes  Abdominal bloating  Breast tenderness  Mood swings | 1  2  3  4  5 |  |
| 18 | Problems faced during menstruation | Cramp, back pain and discomfort  Loss of appetite , headach and tired  Full of abdomen, difficult to walk | 1  2  3 |  |
| 19 | Remedial measures for problems  during menstruation* Multiple response possible | Maintain personal  Hygiene  Take rest  Drink lots of water / eat  Fruits  Exercise  Take medicines | 1  2  3  4  5 |  |
| 20 | Miss class due to menstruation (last 3 months) | Yes  No | 1  2 |  |
| 21 | If yes, how often | One day every cycle  Two days every cycle  Three days every cycle  More than three days every cycle | 1  2  3  4 |  |

**SECTION IV: Cultural restriction faced during menstruation**

| **S. N.** | **Questions** | **Response Categories** | **Codes** | **Skip** |
| --- | --- | --- | --- | --- |
| 1 | Do you ever experienced cultural restriction during menstruation | Yes ………  No ……. | 1  2 | If yes move to question no 2 |
| 2 | Restriction during menstruation   1. Cannot visit temple 2. Cannot attend religious function 3. Cannot do household puja (blessing) 4. Cannot touch male family member 5. Cannot cook food or enter inside kitchen 6. Cannot go outside as much as normal 7. Cannot eat food or drinks of their choice 8. Cannot sleep in the same bed with others 9. Cannot sleep in the household as others | Yes No  Yes No  Yes No  Yes No  Yes No  Yes No  Yes No  Yes No  Yes No | 1 2  1 2  1 2  1 2  1 2  1 2  1 2  1 2  1 2 |  |

**THANK YOU for participating in this survey.**

**If you have any queries regarding the study, please feel free to contact on 9845069800**

**Signature of the participants: ……………**

**c'g'R5]b**

**cg'R5]b ! M– sG;]G6 kmf/fd**

| **;xefuLx?sf nflu k\|ZgfjnL**  gd:t] d]/f] gfd ufoqL vgfn xf] . d :s"n ckm klJns x]Ny, lrtjg d]l8sn sn]h, lqe'jg ljZjljBfno s}nfzgu/, lrtjgdf sfo{/t 5' . d lszf]/Lx?sf] dfl;s wd{, ;/;kmfO{ Joj:yfkg ;DjlGw ljifodf cWoog ub}{5' . o;} qmddf d tkfO{x?;Fu s]lx k\|Zgx? ;f]Wg rfxG5' . o;sf] nflu tkfO{x?n] dnfO{ sl/j #) ldg]6sf] ;do lbg'kg]{5 . tkfO{n] k\|bfg ug'{ ePsf] hfgs/fL dfl;s wd{, :jf:Yo sfo{qmd of]hgf / sfo{Gjogsf] nflu pkof]uL x'g] ck]Iff /flvPsf]5 . tkfO{n] k\|bfg ug'{ ePsf] hfgsf/Lx? s]jn cWoogsf nflu dfq k\|of]u x'g]5 / tkfO{n] lbPsf] hfgsf/L uf]Ko /flvg]5 . olb tkfO{ ;Fu o; cWoogsf] af/]df s]lx k\|Zgx? ug'{5eg] ;f]Wg ;Sg'x'g]5 .  =========================================================================================================================  =========================================================================================================================  s] tkfO{ cWoogdf efu lng rfxg'x'G5 .  rfxG5' ==================================  rfxlbg ===================================  olb rfxg'x'G5 eg] cGtjftf{ ;'? ub{5' . |
| --- |

**v08 ! M ;fdflhs tyf hg;fVoLo ljj/0f**

| **qm=;=** | **k\|Zg** | **pQ/** | **sf]8{** | **5f]8g';** |
| --- | --- | --- | --- | --- |
| ! | pd]/, xfn k'/f ePsf] pd]/ |  |  |  |
| @ | hft÷kl/jf/sf] hfltotf | blnt,  hghflt,  dw]zL,  d'lZnd,  jXfd0f÷If]qL,  7s'/L ÷ ;Gof;L  cGo ======================== | 1  2  3  4  5  6 |  |
| # | wd{ | lxGb'  jf}l4i6  d'lZnd  lqmlZog  cGo ======================== | 1  2  3  4  5 |  |
| $ | lzIff -sIff_ |  |  |  |
| ^ | a;f]af; | zx/L  ufpF | 1  2 |  |
| & | a'afsf] lzIff | lg/If/  ;fIf/  k\|yflds  dfWolds  pRr dfWolds  :gfts jf ;f] eGbf dfly | 1  2  3  4  5  6 |  |
| * | cfdfsf] lzIff | lg/If/  ;fIf/  k\|yflds  dfWolds  pRr dfWolds  :gfts jf ;f] eGbf dfly | 1  2  3  4  5  6 |  |
| ( | lblbjxLgsf] ;+Vof | !  ! eGbf j9L | 1  2 |  |
| !) | :s"nsf] k\|sf/ | ;/sf/L  lghL | 1  2 |  |
| !! | :s'n /x]sf] :yfg | zx/sf] glhs  zx/af6 6fF9f | 1  2 |  |
| !@ | kl/jf/sf] lsld; | Psn kl/jf/  ;+o'Qm kl/jf/ | 1  2 |  |
| !# | a'afsf] k\|d'v Joj;fo | s[lif  Joj;fo  gf]s/L  j}b]lzs /f]huf/  Hofnfbf/L  cGo | 1  2  3  4  5  6 |  |
| !$ | cfdfsf] k\|d'v Joj;fo | s[lif  Joj;fo  gf]s/L  j}b]lzs /f]huf/  Hofnfbf/L  cGo | 1  2  3  4  5  6 |  |
| !% | klxnf] k6s dlxgfjf/Lsf] pd]/ | !)–!# jif{  !$–!^ jif{  !&–@) jif{ | 1  2  3 |  |
| !^ | ldNg] ;fyLx?sf] ;+Vof | 5}g  ! hgf  ! eGbf j9L | 1  2  3 |  |
| !& | s] tkfO{ dlxgfaf/L ;/;kmfO{ ;DjlGw sfo{qmddf ;xefuL x'g' ePsf] 5 . | 5  5}g | 1  2 |  |
| !* | s] tkfO{sf] kl/jf/sf] dfl;s cfDbfgLn] vfgf / b}lgs lqmofsnfk ug{ k\|fKt 5 | k\|of{Kt  ck\|ofKt | 1  2 |  |

**v08 @ M dlxgfjf/L ;/;kmfO{ ;DjlGw 1fg**

| **qm=;=** | **k\|Zg** | **pQ/** | **sf]8{** | **5f]8g';** |
| --- | --- | --- | --- | --- |
| ! | s] tkfO{ klxnf] k6s dlxgfaf/L x'g' eGbf klxn] g} dlxgfjf/Lsf] af/]df hfgsf/ x'g'x'GYof] | lyP  lyOg | 1  2 |  |
| @ | olb hfgsf/L lyof] eg] sxfFaf6 hfgsf/L k\|fKt eof] . | cfdfaf6  lblbaf6  efph'af6  ;fyLx?af6÷cfkmGtaf6  lzIfs÷lzlIfsfaf6  ;~rf/dfWodaf6-lstfj ÷ klqsf, :jf:Yo ;DjlGw lstfj, /]l8of] jf 6]lnlehg_ | 1  2  3  4  5  6 |  |
| # | s] tkfO{n] kfPsf] hfgsf/L pknAwL d'n's lyof] . | lyof]  lyPg | 1  2 |  |
| $ | klxnf] k6s dlxgfaf/L tkfO{ sxfF x'g' ePsf] lyof] . | 3/df  :s"ndf  cGo | 1  2  3 |  |
| % | klxnf] k6s dlxgfjf/L x'Fbf tkfO{sf] k\|ltlqmof s:tf] /x\of] | /f]P  8/fP  nfh nfUof]  v'zL eP | 1  2  3  4 |  |
| ^ | dlxgfaf/L s] xf] < | ;fdfGo k\|lqmof xf]  zl//af6 g/fd\|f] /ut hfg]  cGo -kfk, eujfgsf] sf/0fn]_  yfxf 5}g | 1  2  3  4 |  |
| & | s] dlxgfaf/L ue{jtL x'g] s'/f;Fu ;DjlGwt | 5  5}g | 1  2 |  |
| * | dlxgfaf/L x'g'sf] sf/0f | xdf]{g  /f]u  yfxf 5}g | 1  2  3 |  |
| ( | dlxgfjf/L x'Fbf sxfFaf /ut jU5 | kf7]3/  lk;fj y}nL  lk;fj 3/sf] d'v  k]6 | 1  2  3  4 |  |
| !) | ;fdfGotof dlxgfjf/Ldf slt lbg;Dd /ut jUb5 | < # lbg  # b]lv % lbg  > % lbg | 1  2  3 |  |
| !! | gd{n dlxgfaf/L slt lbgdf cfpF5 | @* lbg  @) lbg  #) lbg  #% lbg | 1  2  3  4 |  |
| !@ | ;]g]6/L Kof8 k\|of]u ug'{sf] sf/0f s] xf] < | dlxgfjf/Lsf] /ut / :jf:Yosf] ;Gt'ngsf] nflu  b'vfO{ sd ug{sf] nflu  gg'jfpgsf] nflu  yfxf 5}g | 1  2  3  4 |  |
| !# | k\|uf]u ul/Psf] Kof8 slt ;dodf kl/jt{g ug'{ k5{ . | x/]s 306fdf  x/]s $ b]lv ^ 306fdf  lbg lbg} | 1  2  3 |  |
| !$ | k\|of]u ePsf] Kof8 s;/L Joj:yfkg ug]{ | hnfP/  uf8]/  ˆofs]/  b'j} ! / @ | 1  2  3  4 |  |
| !% | dlxgfaf/L x'g' eGbf klxn} b]lvg] k\|d'v ;d:ofx? s] s] x'g * jx' pQ/ ;Dej 5 | 6fpsf] b'Vg]  sJhLot÷lbzf nfUg]  k]6df UofF;  :tgdf b'vfO{  dfgl;s kl/jt{g | 1  2  3  4  5 |  |
| !^ | dlxgfaf/L x'Fbf b]lvg] k\|d'v ;d:ofx? s] s] x'g * jx' pQ/ ;Dej 5 | jfpl8g], 9fF8 b'Vg] / c;xh dxz'; x'g]  ef]s gnfUg], 6fpsf] b'Vg] / ysfO{ nfUg]  k]6 e/LP h:tf] x'g] / lx8Fg ufX\|f] x'g] | 1  2  3 |  |
| !& | dlxgfaf/L x'Fbf b]lvPsf ;d:ofx? ;dfwfg ug]{ tl/sfx? s] s] x'g * jx' pQ/ ;Dej 5 | zfl//Ls :jf:Yo Joj:yfkg  cf/fd ug]{  k\|z:t dfqfdf kfgL lkpg]÷  kmnkm'n vfg  zfl/l/s Jofod ug]{  cf}ifwL k\|of]u ug]{ | 1  2  3  4  5 |  |

**v08 # M dlxgfjf/L ;/;kmfO{ Joj:yfkg ;DjlGw k|of]u**

| **qm=;=** | **k\|Zg** | **pQ/** | **sf]8{** | **5f]8g';** | |
| --- | --- | --- | --- | --- | --- |
| ! | dlxgfaf/L Joj:yfkg ug{ ;fdfGoto tkfO{ s] ;fdfu\|L k\|of]u ug'{x'G5 . | sk8f  ;]g]6/L Kof8-ˆofSg ldNg]_  s'g} klg ;fdu\|L k\|of]u ubL{g  ;]g]6/L Kof8 -k'g k\|of]u ug{ ldNg]_ | 1  2  3  4 |  | |
| @ | olb tkfO{n] 5fGg kfpg' eof] eg] dlxgfjf/L Joj:yfkgsf] nflu s'g ;fdfu\|L k\|of]u ug{ ?rfpg' x'G5. | ;]g]6/L Kof8-ˆofSg ldNg]_  s'g} klg ;fdu\|L k\|of]u ubL{g  ;]g]6/L Kof8 -k'g k\|of]u ug{ ldNg]_  sk8f -k'gM k\|of]u ug{ ldNg]_  cGo -k'gM k\|of]u ug{ ldNg] ;]g]6/L Kof8, l6=;'= k]k/_ | 1  2  3  4  5 |  | |
| # | s] tkfO{ ;]g]6/L Kof8 cfkm\gf] 3/af6 @) ldg]6sf] b'/Ldf lsGg ;Sg'x'G5 . | ;S5'  ;lSbg | 1  2 |  | |
| $ | s] tkfO{n] ;]g]6/L Kof8s -ˆofSg] k\|of]u ug'{ ePsf] 5_ | 5  5}g | 1  2 | olb 5}g eg] k\|Zg g+= * df hfg' xf]; . | |
| % | olb 5 eg] slt dfqfdf k\|of]u ug'{x'G5 . | k\|fo  slxn] sfxL  lj/n} | 1  2  3 |  | |
| ^ | ;]g]6/L Kof8 s;n] lsGglbg] u/]sf] 5 . | cfkm}n]  cfkmGtn] | 1  2 |  | |
| & | sxfFaf6 lsGg] u/]s]f 5 . | ahf/sf] k;naf6  ufpF3/sf] k;naf6  cGo -:s"n af6 k\|fKt, cf}iflw k;n _ | 1  2  3 |  | |
| * | olb ;]g]6/L Kof8 k\|of]u gug]{ jf lj/n} k\|of]u ug]{ u/]sf] 5 eg] To;sf sf/0fx? s] s] x'g * jx' pQ/ ;Dej 5 | ;lhn} gkfP/  sk8fsf] Kof8 ;lhnf] nfu]/  dxuf] eP/  s;/L k\|of]u ug]{ eGg] yfxf geP/  lsGg hfg nfh nfu]/  ˆofSg ufX\|f] eP/  cGo-slxn] g;'g]sf], PnhL{_ | 1  2  3  4  5  6 |  | |
| ( | dlxgfaf/Lsf] a]nfdf tkfO{ sltsf] g'jfpg' x'G5 . * jx' pQ/ ;Dej 5 | klxnf] lbg  bf];\|f] lbg  t];\|f] lbg  rf}yf} lbg  kfFrf} lbg  5}7f}+ lbg  x/]s lbg | 1  2  3  4  5  6 |  | |
| !) | dlxgfaf/L ePsf] a]nf g'xfpFbf tkfO{ slt dfqfdf ;fj'gsf] k\|of]u ug'{x'G5 . | ;w} k\|of]u u5' .  slxn] sfxL k\|of]u u5' .  slxNo klg k\|of]u ulb{g . | 1  2  3 |  | |
| !! | dlxgfaf/L ePsf] a]nf tkfO{ sltdfqfdf cfkm\gf] uf]Ko c+u ;kmf ug'{x'G5 . | ;w} ;kmf u5' .  slxn] sfxL ;kmf u5' .  slxNo klg ;kmf ulb{g . | 1  2  3 |  | |
| !@ | dlxgfaf/L ePsf] a]nfdf tkfO{ slt dfqfdf cf/fd ug'{x'G5 . | ;fdfGo eGbf j9L  ;fdfGo h:t}  ;fdfGo eGbf sd  yfxf ePg | 2  3  4 |  | |
| !# | s] tkfO{ k'g k\|of]u ug]{ Kof8 tyf sk8fx? ;kmf ubf{ uf]Kotf Joj:yfkg ug'{x'G5 . | u5'{  ulb{g  d k'gM k\|of]u ug]{ Kof8 tyf sk8f k\|of]u ulb{g . | 1  2  3 |  | |
| !$ | k'gM k\|of]u ug]{ sk8f tyf Kof8snfO{ sxfF ;'sfpg' x'G5 . | 3/ eGbf jflx/  3fFd nfu]sf] 7fpFdf  c? sk8fx? hxfF ;'sfOG5 ToxL ;'sfpg]  3fd gnfu]s]f 7fpFdf  ;'sfpbf cGo sk8fn] 5f]k]/ ;'sfp5'  cGo | 1  2  3  4  5  6 |  | |
| !% | s] tkfO{ Kof8 kl/jt{g u/]kl5 xft w'g'x'G5 | w'G5'  w'Gg | 1  2 |  | |
| !^ | k\|of]u ePsf] Kof8 sxfF KofSg] ug'{ ePsf] 5 . | 8i6ljgdf  jfl/df ˆofSg] u/]s]f 5'  gu/kflnsf cyjf ufpFkflnsfsf] kmf]x/df kmfNg] u/]s]f 5' .  cGo | 1  2  ३  ४ |  | |
| !& | dlxgfaf/L x'g' eGbf klxn] tkfO{df b]lvg] k\|d'v ;d:ofx? s] s] x'g * jx' pQ/ ;Dej 5 | 6fpsf] b'Vg]  sJhLot÷lbzf nfUg]  k]6df UofF;  :tgdf b'vfO{  dfgl;s kl/jt{g | 1  2  ३  ४ |  |  |
| !* | dlxgfaf/L x'Fbf tkfO{nfO{ b]lvg] k\|d'v ;d:ofx? s] s] x'g * jx' pQ/ ;Dej 5 | jfpl8g], 9fF8 b'Vg] / c;xh dxz'; x'g]  ef]s gnfUg], 6fpsf] b'Vg] / ysfO{ nfUg]  k]6 e/LP h:tf] x'g] / lx8Fg ufX\|f] x'g] | 1  2  ३ |  |  |
| !( | dlxgfaf/L x'Fbf b]lvPsf ;d:ofx? ;dfwfg ug]{ tkfO{ s] s] tl/sfx? ckgfpg' x'G5 * jx' pQ/ ;Dej 5 | zfl//Ls :jf:Yo Joj:yfkg  cf/fd ug]{  k\|z:t dfqfdf kfgL lkpg]÷  kmnkm'n vfg  zfl/l/s Jofod ug]{  cf}ifwL k\|of]u ug]{ | 1  2  3  4  5 |  |  |
| @) | s] tkfO{n] dlxgfjf/L ePsf] sf/0f kl5Nnf] # dlxgfdf cfkm\gf] Sn;; 5f]8\g' ePsf] 5 < | 5  5}g | 1  2 |  |  |
| @! | olb 5 eg] slt | ! lbg x/]s k6s dlxgfjf/L x'Fbf  @ lbg x/]s k6s dlxgfjf/L x'Fbf  # lbg x/]s k6s dlxgfjf/L x'Fbf  # lbg eGbf j9L x/]s k6s dlxgfjf/L x'Fbf | 1  2  3  4 |  |  |

**v08 $ M ;f+:s[lts÷;fdflhs k|ltjGw**

| **qm=;=** | **k\|Zg** | **pQ/** | **sf]8{** | **5f]8g';** |
| --- | --- | --- | --- | --- |
| ! | s] tkfO{n] dlxgfjf/L ePsf] a]nf ;f+:s'ls ÷;fdflhs k\|ltaGw cg'ej ug'{ ePsf] 5 < | 5  5}g | !  @ | olb 5 eg] k\|Zg g+=@ df hfg'xf];\ |
| @ | dlxgfjf/L ePsf] a]nf cg'ej u/]sf k\|ltaGwx?  != dlGb/ leq hfg gkfpFg]  @= wfld{s sfo{qmddf ;xeflu x'g gkfpg]  #= 3/sf] k"hf{ kf7df ;xeflu x'g gkfpg]  $= kl/jf/sf] k'?if ;b:onfO{ 5'g gkfpg]  %= vfg ksfpg jf efG;fdf l5g{ gkfpg]  ^= ;fdfGo c? a]nf h:tf] u/]/ aflx/ lg:sg gkfpg  &= cfˆgf] OR5f cg';f/sf] vfgf tyf lkpg] kbf{y lkpg gkfpg]  *= 3/df cfkm' ;'lt/x]sf] a]8df ;'Tg gkfpg]  (= 3/sf cGo ;b:o ;Fu ;'Tg gkfpg | xf] xf]Og  xf] xf]Og  xf] xf]Og  xf] xf]Og  xf] xf]Og  xf] xf]Og  xf] xf]Og  xf] xf]Og  xf] xf]Og | ! @  ! @  ! @  ! @  ! @  ! @  ! @  ! @  ! @ |  |

o; ;j]{If0fdf efu lng'ePsf]df wGofjfb

| **qm=;=** | **cjnf]sg** | **r]s ln:6** | **sf]8{** | **5f]8g';** |
| --- | --- | --- | --- | --- |
| ! | s] 6\jfOn]6sf] r's'nx? sfd ug]{ sfd ug]{ cj:yfdf 5g\ | 5 5}g | ! @ | olb 5 eg] k\|Zg g+=@ df hfg'xf];\ |
| @ | ljBfnodf s'n 6\jfO{n]6sf ;+Vofx? | ========================================= |  |  |
| # | s] s]6Lx?sf] nflu 5'§} 6\jfOn]6 5 < | 5 5}g | ! @ |  |
| # | s] ljBfnosf] 6\jfOn]6 jf 6\jfOn]6sf] jl/kl/ 8i6ldg /flvPsf] 5 < | 5 5}g | ! @ |  |
| $ | s] /flvPsf] 8i6ljgx? jGb lsl;dsf 5g\ < | 5 5}g | ! @ |  |
| % | s] 8i6ljgx? ;dodf ;kmf tyf vfnL ug]{ u/LPsf] 5 < | 5 5}g | ! @ |  |
| ^ | s] ljBfnodf kfgLsf] /fd\|f] Joj:yf 5 < | 5 5}g | ! @ |  |
| & | s] ljBfnodf ;fd'gsf] Joj:yf /fd\|f] 5 < | 5 5}g | ! @ |  |
| * | s] 6\jfOn]6 ;do ;dodf ;kmf u/LG5 < | 5 5}g | ! @ |  |
| ( | s] 6\jfOn]6x?df g/fd\|f] uGw cfpF5 < | 5 5}g | ! @ |  |
| !) | s] ljBfnosf] 6\jfOn]6x?df lbzf dfly dfly b]lvG5 < | 5 5}g | ! @ |  |
| !! | s] ljBfno leq :jf:YosdL{x?sf] Joj:yf ul/Psf] 5 < | 5 5}g | ! @ |  |
| !@ | s] ljBfnodf ljBfyL{x?nfO{ cfjZos k/]sf] j]nfdf cfktsflng Kof8 pknAw u/fpg] Joj:yf 5 < | 5 5}g | ! @ |  |

olb tkfO{ ;Fu cWoog ;DaGwL s'g} k|Zgx? 5g eg] s[kof (*$%)^(*)) df ;Dk{s ug{ glxrlsrfpg' xf]nf

;xeflusf] x:tfIf/ M =================================
