# Supplementary material for: Menstruation hygiene management among secondary school students of Chitwan, Nepal:a cross-sectional study
Source: BMC Womens Health. 2023 Jul 26;23:395. doi: 10.1186/s12905-023-02494-x (PMC10373230; doi:10.1186/s12905-023-02494-x)
Supplement: Supplementary file 2 — Additional File 2: Nepal Health Research Council [file 12905_2023_2494_MOESM2_ESM.pdf]

Government of Nepal
Nepal Health Research Council (NHRC)
Estd. 1991

Ref. No.: 2785

Date: 7 April 2021

Ms. Gayatri Khanal

Principal Investigator

Chitwan Medical College

Chitwan

Ref: Approval of research proposal

Dear Ms. Khanal,

This is to certify that the following protocol and related documents have been reviewed and granted approval by the Expedited Review Sub-Committee for implementation.

**ERB Protocol Registration No.**	139/2021 P		**Sponsor Protocol No**	NA	
**Principal Investigator/s**	Ms. Gayatri Khanal		**Sponsor Institution**	NHRC Grant	
**Title**	Menstruation Hygiene management among secondary school students of Chitwan, Nepal- Pre –Post Study Design				
**Protocol Version No**	NA		**Version Date**	NA	
**Other Documents**	1. Data collection tools<br>2. Acceptance letter from the study sites		**Risk Category**	Minimal risk	
**Expedited Review**	Proposal	√	**Duration of Approval**<br>7 April 2021 to<br>7 April 2022	**Frequency of continuing review**	
	Amendment				
	Re-submitted				
	**Meeting Date:** 30 March 2021				
**Total budget of research**	**NRs 1,50,000.00**				
**Ethical review processing fee**	**Waiver as the researcher had received by NHRC Grant**				

Investigator Responsibilities :

- Any amendments shall be approved from the ERB before implementing them
- Submit progress report every 3 months
- Submit final report after completion of protocol procedures at the study site
- Report protocol deviation / violation within 7 days

Tel: +977 1 4254220, Fax: +977 1 4262469, Ramshah Path, PO Box: 7626, Kathmandu, Nepal
Website: http://www.nhrc.gov.np, E-mail: nhrc@nhrc.gov.np

Ref. No.: 2785

- Comply with all relevant international and NHRC guidelines
- Abide by the principles of Good Clinical Practice and ethical conduct of the research

If you have any questions, please contact the Ethical Review M & E Section at NHRC.

Thanking you,

Dr. Pradip Gyanwali
Member-Secretary
(Executive Chief)
