# Supplementary material for: Menstruation hygiene management among secondary school students of Chitwan, Nepal:a cross-sectional study
Source: BMC Womens Health. 2023 Jul 26;23:395. doi: 10.1186/s12905-023-02494-x (PMC10373230; doi:10.1186/s12905-023-02494-x)
Supplement: Supplementary file 3 — Additional File 3: Total number of participants selected from each selected school [file 12905_2023_2494_MOESM3_ESM.docx]

**Total number of participants selected from each selected school**

| **Name of the School** | **Total number students (1237)** | **Number of students selected** |
| --- | --- | --- |
| Bharatpur ma vi | 55 | 18 |
| Prembasti School | 70 | 22 |
| Sharadpur School | 93 | 30 |
| Narayani Ma. Bi | 72 | 23 |
| Amber Everest | 44 | 15 |
| Hill bird | 60 | 20 |
| Small heaven | 70 | 22 |
| Sunrise School | 78 | 25 |
| Ankuram Ma vi | 48 | 15 |
| Nepal adarsha Sikshya Sadan | 72 | 23 |
| Chitwan Ma. Bi | 70 | 22 |
| Dawn Academy | 71 | 24 |
| Little flower | 65 | 21 |
| Holy Vision | 38 | 12 |
| Gitanagar | 60 | 20 |
| Sukranagar | 32 | 11 |
| Sardanagar | 46 | 15 |
| Kamal devi | 53 | 17 |
| Sky rider | 50 | 16 |
| Ratnanagar | 40 | 13 |
| Pithuwa School | 50 | 16 |
| Total Number | 1237 | 400 |
